# The Inhibitory Effect of *Agastache rugosa* Essential Oil on the Dental Biofilm

**DOI:** 10.3390/molecules29204907

**Published:** 2024-10-16

**Authors:** Eun Sook Kim, Bog-Im Park, Young-Hoi Kim, Jooyi Kang, Yong-Ouk You

**Affiliations:** 1 Institute of Biomaterials and Implant, College of Dentistry, Wonkwang University, Iksan 54538, Republic of Korea; bacteria@wku.ac.kr; 2 Department of Oral Biochemistry, School of Dentistry, Wonkwang University, Iksan 54538, Republic of Korea; parkbogim@hanmail.net (B.-I.P.); joo2queen@hanmail.net (J.K.); 3 Department of Food and Nutrition, School of Food, Kunsan National University, Kunsan 54150, Republic of Korea; 4 Department of Food Science and Biotechnology, Jeonbuk National University, Jeonju 54896, Republic of Korea; yhoi1307@hanmail.net; 5 Wonkwang Dental Research Institute, Wonkwang University, Iksan 54538, Republic of Korea

**Keywords:** *Agastache rugosa*, essential oil, biofilm, GC-MS, estragole

## Abstract

This study aimed to identify the inhibitory effect of *Agastache rugosa* essential oil (AREO) on the cariogenic properties of *Streptococcus mutans*, which causes dental caries and dental plaque formation. After extracting the AREO, their effects on the growth and acid production of *S. mutans* were examined. Furthermore, *S. mutans* biofilm formation was observed on the resin teeth surface. The effect on the expression of biofilm-related genes of *S. mutans* was measured using real-time PCR. AREO components were analyzed using gas chromatography (GC) and GC-mass spectrometry (MS). The growth and acid production of *S. mutans* were significantly inhibited at concentrations of 0.02 mg/mL or higher of AREO. At 0.04 mg/mL, inhibition was similar to that of the positive control, 0.1% NaF. AREO suppressed the expression of virulence factors such as *gtfB*, *gtfC*, *gtfD*, *gbpB*, *SpaP*, *brpA*, *relA*, and *vicR* at concentrations of 0.02 mg/mL or higher. As a result of GC and GC-MS analyses, the main components of AREO included estragole, limonene, and β-caryophyllene. These results suggest that *A. rugosa* may be a useful agent for inhibiting the cariogenic properties of *S. mutans*.

## 1. Introduction

Dental caries is a disease that gradually and irreversibly destroys the hard tissues of teeth, such as the enamel, cementum, and dentin [1,2]. Dental caries are prevalent worldwide and are a representative cause of tooth loss in humans. As the incidence tends to increase with increasing sugar consumption and longer life expectancy, effective management measures are urgently needed [3,4].

Dental caries is caused by dental plaque, a type of dental biofilm formed by oral bacteria. A large number of microorganisms are known to exist in dental biofilm. These microorganisms induce dental caries by dissolving the hard tissues of teeth and forming organic acids by metabolizing sugars.

*Streptococcus mutans* is known as one of the causative bacteria that play the most important role in the formation of dental plaque and the induction of dental caries. *S. mutans* is a Gram-positive facultative anaerobic bacterium that grows well even under aerobic conditions, which resides on the tooth surface in the oral cavity and uses carbohydrates, especially glucose and fructose contained in ingested food, to produce organic acids (mainly lactic acid) in the process of metabolism and discharges them outside of the cells. Extracellular organic acids demineralize hard tissues, such as tooth enamel, dentin, and cementum [5,6,7]. Furthermore, glucosyltransferase (GTFase) produced by *S. mutans* catalyzes the synthesis of fructan by fructosyltransferase (FTFase) and glucan, an extracellular polysaccharide, using sucrose as a substrate [1,3]. These adhesive and insoluble glucans and fructans act to firmly adhere *S. mutans* to the tooth surface together with other bacteria and increase the formation of dental plaque. In addition, extracellular polysaccharide reduces the permeability of the dental plaque, preventing the diffusion of acids produced by bacteria in the dental plaque, acting as a barrier against the penetration of saliva, which has a buffering action, and promoting demineralization of the tooth surface by concentrating the acid produced by oral bacteria inside the dental plaque [4,8,9,10].

Fluoride compounds and chlorhexidine are used to inhibit the formation of dental microfilm, while vaccine development is also being carried out. However, fluoride compounds are toxic to oral cells at more than 80 ppm [11]. Chlorhexidine has an unpleasant taste, increases the acquired pellicle, and discolors teeth yellow when used for a long time [12,13].

Recently, studies have been conducted to suppress the formation of dental plaque using vaccines, but they are not sufficiently effective due to high development costs and low efficacy. The fact that dental caries due to dental plaque is still a major cause of tooth loss is evidence that the existing methods are not sufficiently effective. Therefore, there is a demand for the development of a more effective and practical method for inhibiting dental plaque.

Many studies have analyzed *Agastache rugosa* (Fisch. & C.A.Mey.) O. Kuntze essential oil (AREO) and reported the presence of various volatile organic compounds. Estragole, also known as methyl chavicol, is the main component of AREO [14,15,16]. The components were also analyzed, and nine compounds were identified as follows: menthone, isomenthone, dihydrocarbone, anethole, vanillin, eugenol, methyl eugenol, β-caryophyllene, and β-caryophyllene oxide [17]. Physiological activities include antibacterial, antifungal, antiviral, antimutagenic, anticancer, nematicidal, insecticidal, antioxidant, and Alzheimer’s alleviating activities [18].

In particular, AREO has shown strong antibacterial activity against *Staphylococcus aureus* [17]. The essential oil extracted from *A. rugosa* leaves has been reported to have an antibacterial effect against *E. coli* (MIC, 9.4 µg/mL). The essential oil of *A. rugosa* flowers inhibits biofilm activity and has shown similar or stronger activity against *S. aureus* (MIC, 21 μg/mL) than penicillin [19]. AREO also inhibits *Escherichia coli* O157:H7 ATCC 43895, which causes hemorrhagic nephrotic syndrome and enteritis, and *Salmonella typhimurium* ATCC 7988, which causes intestinal diseases, such as typhoid in humans, in a concentration-dependent manner. AREO also exhibited antifungal activity against *Aspergillus niger*, *Candida albicans*, and *Cryptococcus neoformans* [16].

The chloroform and ethyl acetate solvent fractions of *A. rugosa* extracted with hot water have been reported to have strong antibacterial activity against 15 types of microorganisms. As a result of gas chromatography-mass spectrometry (GC-MS) analysis, eugenol and its isomers, which have structural similarities with estragole, which is a strong antibacterial substance with a large number of phenolic compounds, were found to be predominant. The chloroform fraction contained 18.1% alkanes and 13.5% ketones, as well as terpenes, acids, and phenols [20].

Although many studies have reported that AREO exhibits antibacterial activity, a few studies have investigated the effects of *A. rugosa* on *S. mutans*, the causative agent of dental plaque formation. Therefore, this study aimed to observe the inhibition of *S. mutans* growth, acid production, and virulence gene expression by AREO, as well as its biofilm formation inhibitory effect on *S. mutans*.

## 2. Results

### 2.1. Inhibitory Effect on the Growth of S. mutans

**Figure 1:**
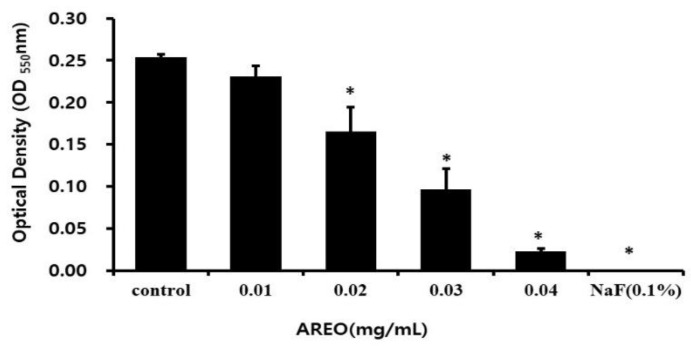
The growth of *S. mutans* inhibitory effect of AREO. The degree of inhibitory activity was observed in the presence of AREO at concentrations of 0.01, 0.02, 0.03, and 0.04 mg/mL. In addition, 0.1% NaF was used as a positive control. Significance was determined at * *p* < 0.05 compared to the control group.

The antibacterial activity of AREO against *S. mutans* was observed. AREO was added at concentrations of 0.01, 0.02, 0.03, and 0.04 mg/mL, and *S. mutans* was inoculated and cultured. The growth rates were 91.1%, 65.4%, 38.2%, and 9.16%, respectively, compared to the 100% growth rate of the control group. At a concentration of 0.04 mg/mL, the growth of *S. mutans* was inhibited similarly to the positive control with the addition of 0.1% NaF (Figure 1).

### 2.2. Inhibitory Effect on Adhesion to Saliva-Coated Hydroxyapatite (S-HA)

**Figure 2:**
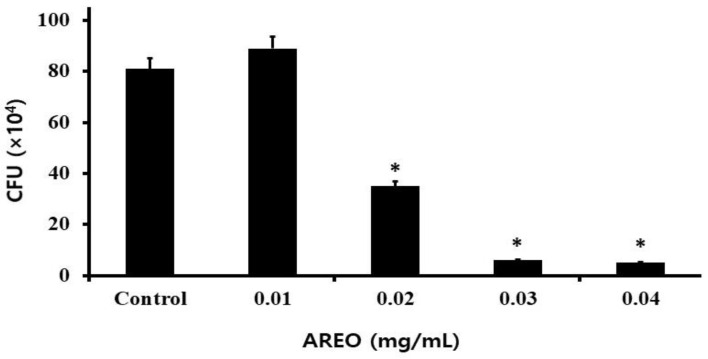
Effect of AREO on the levels of *S. mutans* (CFU). *S. mutans* was inoculated into BHI broth with various concentrations of AREO and incubated for 24 h. The amounts of *S. mutans* that adhered to S-HA beads that were treated with various concentrations of AREO are shown. When treated with 0.01–0.04 mg/mL of AREO, adherence was significantly repressed. Each value is expressed as the mean ± SD. Significance was determined at * *p* < 0.05 compared to the control group.

The adhesion to S-HA of *S. mutans* by AREO was studied, and *S. mutans* was found to be significantly suppressed in a dose-dependent manner. After confirming the effect of inhibiting adhesion to S-HA at an AREO concentration of 0.01–0.04 mg/mL, it was found that 81 ± 4.24 (×10^4^) CFU/mL adhered in the control group. At each concentration and with 89 ± 9.57 (×10^4^) CFU/mL, 35 ± 1.22 (×10^4^) CFU/mL, 6 ± 2.12 (×10^4^) CFU/mL, and 5 ± 1.22 (×10^4^) CFU/mL adhered, respectively, showing 109.9%, 46.2%, 7.5%, and 6.2%, adhesion inhibition rates, respectively, compared to the control group (Figure 2).

**Figure 3:**
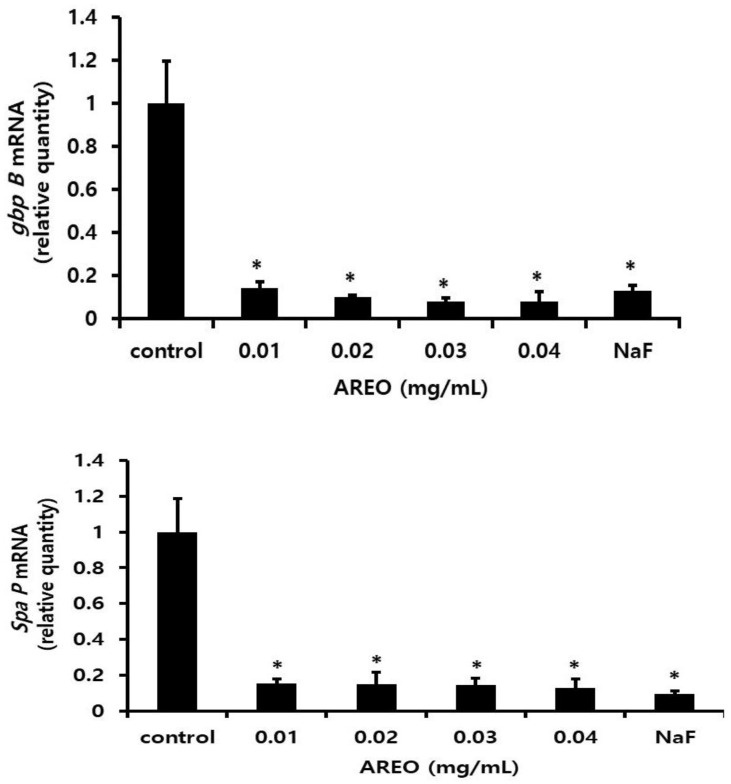
Real-time PCR analysis of multiple genes encoding biofilm formation-associated virulence factors. After culturing *S. mutans* with various concentrations of the AREO component, real-time PCR analysis was performed. Each value is expressed as mean ± standard deviation. Significance was determined at * *p* < 0.05 compared to the control group.

As a result of observing the mRNA expression of *gbpB* and *spaP* to investigate the effect of AREO on the expression of adherence-related genes, real-time PCR analysis showed that *gbpB* and *spaP* mRNA expression was suppressed at concentrations higher than 0.01 mg/mL (Figure 3).

### 2.3. The Inhibitory Effect on S. mutans Biofilm Formation

**Figure 4:**
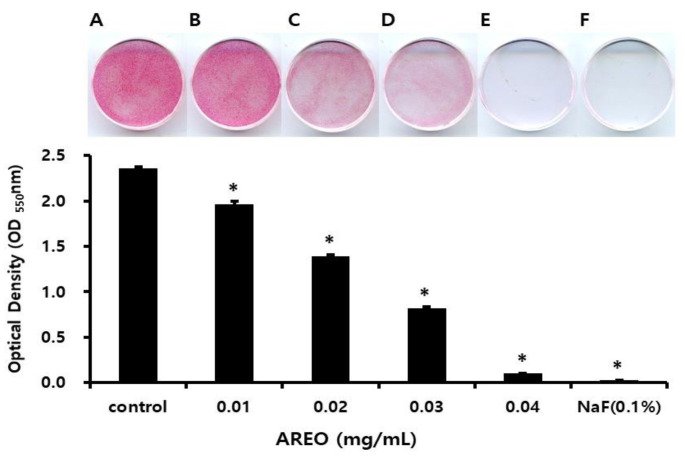
Inhibitory effect of AREO on biofilm formation in *S. mutans*. Safranin staining of *S. mutans* biofilms after treatment with AREO. (**A**) Control, (**B**) 0.01 mg/mL, (**C**) 0.02 mg/mL, (**D**) 0.03 mg/mL, (**E**) 0.04 mg/mL, and (**F**) positive control (0.1% NaF). Significance was determined at * *p* < 0.05 compared to the control group.

**Figure 5:**
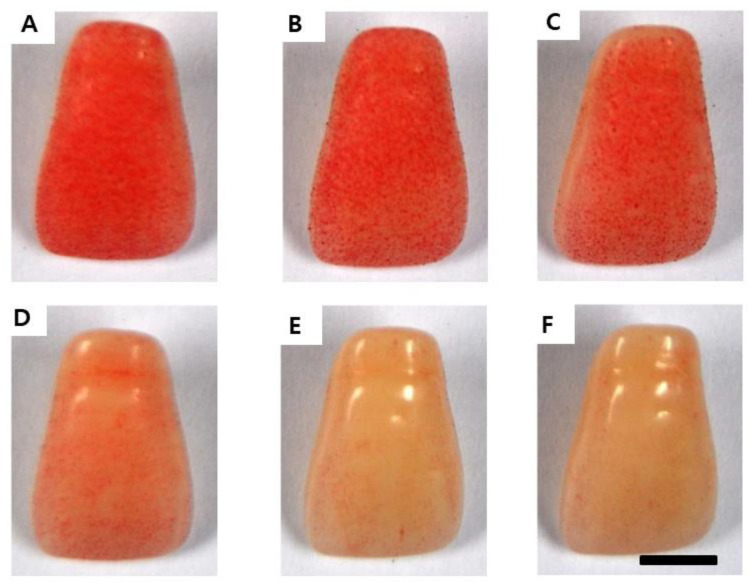
Inhibitory effect of AREO on *S. mutans* biofilm formation on the surface of a resin-based tooth. (**A**) Control, (**B**) 0.01 mg/mL, (**C**) 0.02 mg/mL, (**D**) 0.03 mg/mL, (**E**) 0.04 mg/mL, and (**F**) positive control (0.1% NaF). Bar = 3.5 mm.

**Figure 6:**
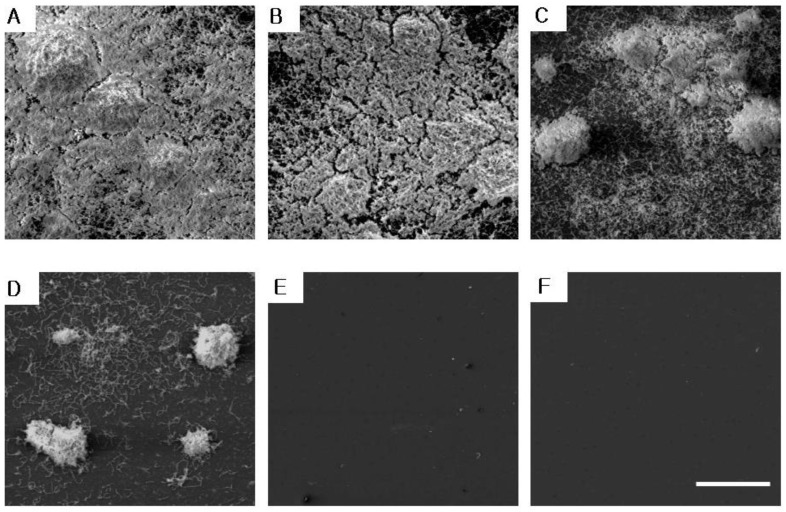
SEM of *S. mutans* biofilms grown in AREO. Biofilm formation was decreased in the presence of AREO at concentrations greater than 0.04 mg/mL. (**A**) Control, (**B**) 0.01 mg/mL, (**C**) 0.02 mg/mL, (**D**) 0.03 mg/mL, (**E**) 0.04 mg/mL, and (**F**) positive control (0.1% NaF). Bar = 50 μm.

Figure 4 and Figure 5 shows the experimental results of the inhibitory effect of AREO on *S. mutans* biofilm formation. A large amount of *S. mutans* biofilm was formed in the control group without AREO, and as the AREO concentration increased, *S. mutans* biofilm formation was suppressed. These experimental results were similar to those observed using an electron microscope (Figure 6). In the control group, a large amount of *S. mutans* biofilm was formed, and as the AREO concentration increased, the formation of the *S. mutans* biofilm was clearly suppressed, even under an electron microscope. In particular, inhibition similar to that of 0.1% NaF, a positive control, was observed at 0.04 mg/mL. In addition, biofilm formation by AREO was suppressed on the surface of artificial teeth, and an excellent effect was observed at a concentration of 0.04 mg/mL or more (Figure 5).

**Figure 7:**
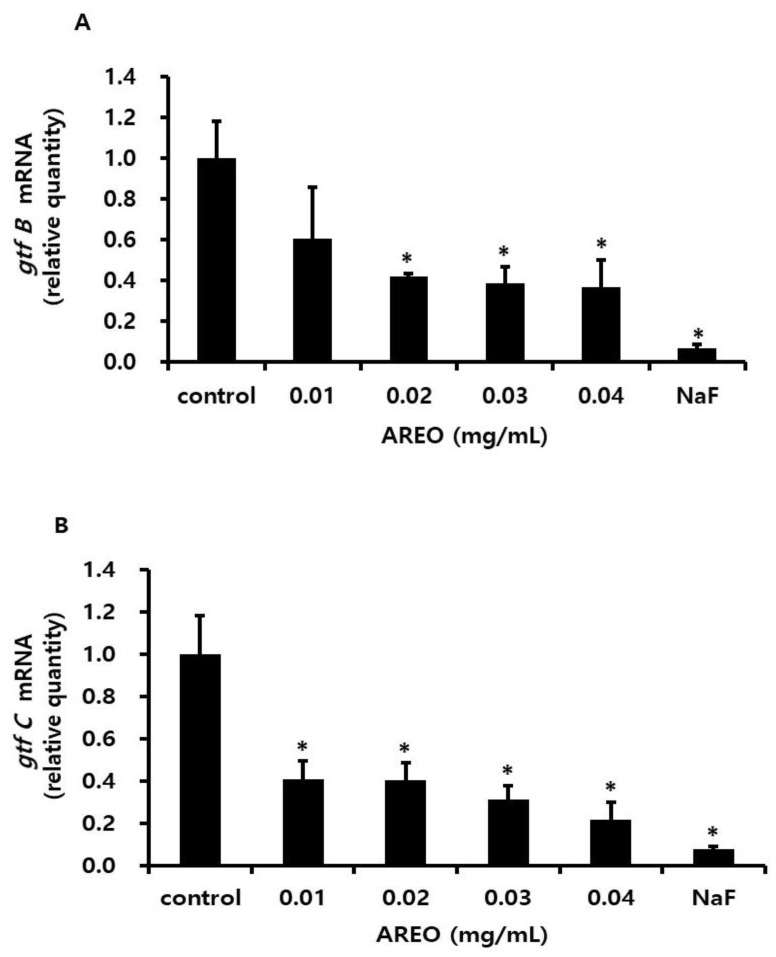

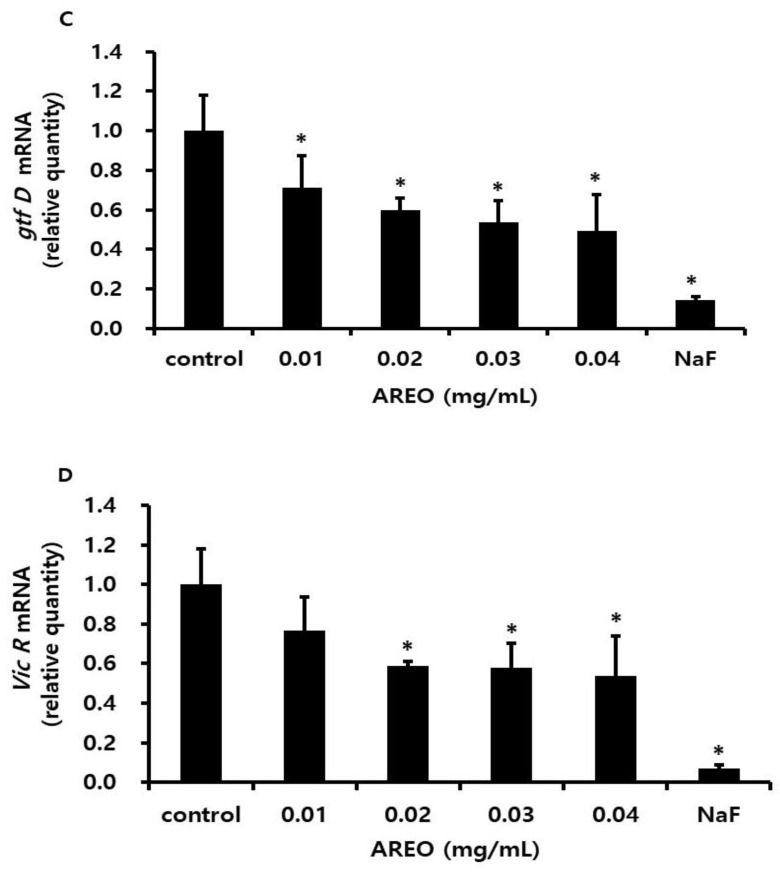
Real-time PCR analysis of multiple genes encoding biofilm formation-associated virulence factors. Relative quantity of *gtfB* (**A**), *gtfC* (**B**), *gtfD* (**C**) and *VicR* (**D**). After culturing *S. mutans* with various concentrations of AREO, real-time PCR analysis was performed. Each value is expressed as the mean ± SD. Significance was determined at * *p* < 0.05 compared to the control group.

It was confirmed using real-time PCR to observe gene expression associated with biofilm formation by AREO. By treatment with AREO concentrations of 0.01–0.04 mg/mL, mRNA levels of *gtfC* and *gtfB* genes were decreased at concentrations of 0.01 mg/mL or higher, and mRNA levels of *gtfB* and *vicR* genes were decreased at concentrations of 0.02 mg/mL or higher (Figure 7).

Regarding the biofilm formation, Figure 4 clearly shows the constant decrease. However, mRNA levels are kept constant over 0.02 mg/mL in Figure 7. These two data are not consistent with each other. Although real-time RT PCR was used to monitor the mRNA levels of key biofilm-related genes, it is well known that there can be a delay between mRNA expression and the corresponding biological activity. This may partially explain the possibility of discrepancy between biofilm formation and mRNA levels. Furthermore, there is a possibility that AREO contributes to biofilm degradation, which requires further investigation in future studies.

### 2.4. Inhibitory Effect on the Acid Production of S. mutans

In order to examine the effect of AREO on the organic acid production of *S. mutans*, *S. mutans* was inoculated into the experimental group, to which AREO was added at concentrations of 0.01, 0.02, 0.03, and 0.04 mg/mL. The mixtures were then cultured for 24 h, followed by measuring the pH with a pH meter. Table 1 shows the results. In the control, the pH before inoculation with bacteria was about 7.38 ± 0.05, but after inoculation and incubation with bacteria, the pH dropped to 5.41 ± 0.05. In the experimental group to which AREO was added, a statistically significant difference was shown at a concentration of 0.02 mg/mL compared to the control group.

We have performed additional experiments to find out whether AREO can neutralize acids. In our study, AREO has no ability to neutralize acid. The pH of the control group cultured with *S. mutans* was 5.31, and the addition of AREO did not result in significant pH changes, indicating that AREO does not have acid-neutralizing properties in this system.

**Figure 8:**
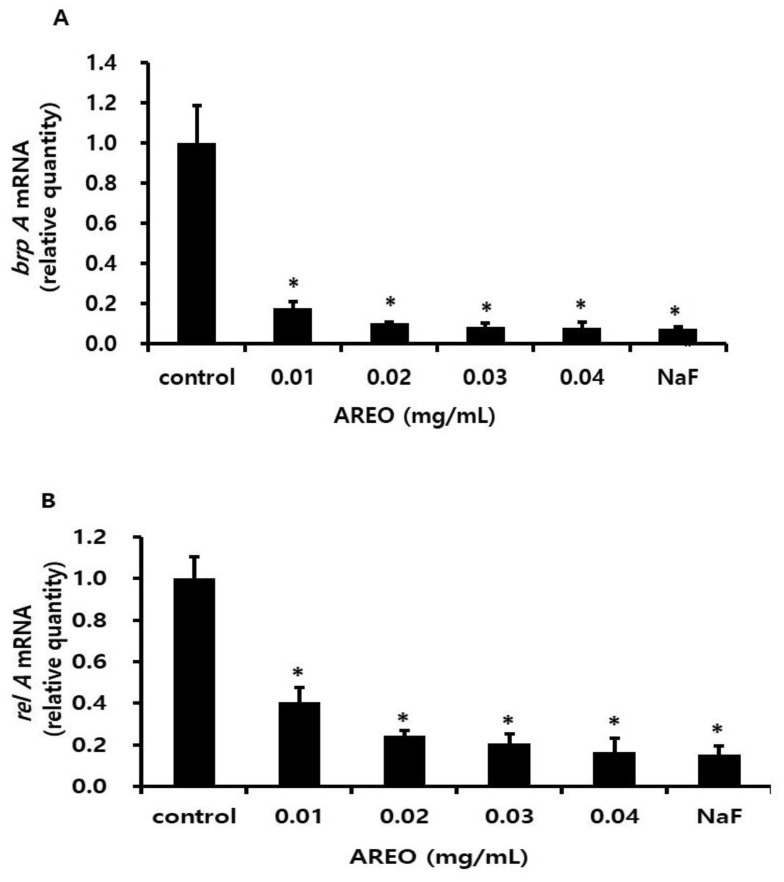
Real-time PCR analysis of multiple genes encoding acid tolerance-related virulence factors. Relative quantity of *brpA* (**A**) and *relA* (**B**). After culturing *S. mutans* with various concentrations of AREO, real-time PCR analysis was performed. Each value is expressed as the mean ± SD. Significance was determined at * *p* < 0.05 compared to the control group.

Gene expression of *brpA* and *relA*, which contribute to acid resistance regulation, was also reduced (Figure 8).

### 2.5. GC and GC-MS Analysis Results of AREO

As a result of analyzing AREO using GC and GC-MS, 43 constituents were identified, accounting for 98.59% of the total essential oil. The major components were estragole (88.69%), *β*-Caryophyllene (2.56%), and limonene (2.29%) (Table 2). Estragole, β-caryophyllene, and limonene are thought to be responsible for the anti-cariogenic properties of AREO. Future research will explore specific roles of major compounds in anti-cariogenic activities.

## 3. Discussion

Research into the prevention and treatment of dental caries, which is the main cause of tooth loss, is ongoing. Nevertheless, the rate of dental caries is increasing, requiring more financial investment and the establishment of appropriate prevention methods. For this reason, continued efforts have been made to develop several natural products using substances that help prevent and treat dental caries and periodontal disease. Many studies have reported that some natural substances inhibit the growth of *S. mutans*, the causative agent of dental caries in the oral cavity, but their mechanisms of action have been reported to be different. *Plagiorhegama dubium* and *Terminalia chebula* extracts have been reported to inhibit the growth and acid production of *S. mutans* [22,23]. *Schisandra chinensis* and *Cinnamomum cassia* Blume extracts have been reported to inhibit *S. mutans* growth and reduce adhesion to S-HA beads [18,24,25]. In addition, propolis [17], *Phellodendron amurense* extract [26], and seaweed extracts, such as funoran [27] and berberine, have also been reported to have antibacterial activity against *S. mutans* [28]. The polyphenols extracted from green tea leaves and the ethyl acetate extract of *Sophora flavescens* inhibit the synthesis of water-insoluble glucan by inhibiting the GTFase of *S. mutans* [29,30], and oolong tea leaf extract inhibits the cell-free GTFase of *S. mutans*, which synthesizes water-insoluble glucan [31]. *Salvia miltiorrhiza*, *Dryopteris crassirhizoma*, and *Platycodon grandiflorus* extracts have been reported to have antibacterial activity against *S. mutans* [32,33,34]. In addition, *Artemisia capillaris*, *Artemisia argyi*, and *Artemisia herba* essential oil extracts have antibacterial effects against *S. mutans* and *Streptococcus sanguinis* [35], and Galla Rhois extract containing tannic acid inhibits dental caries with antibacterial and bactericidal action [36,37].

Therefore, this study aimed to observe the biofilm formation inhibitory effect of AREO, traditionally used for food and medicine. The inhibitory effect of AREO on the growth of *S. mutans*, the causative bacterium of dental caries, was observed at a concentration of 0.02 mg/mL or higher. *Chamaecyparis obtusa* oil at concentrations above 0.05 mg/mL [38] and Brazilian Piperaceae essential oil at concentrations of 0.05–0.5 mg/mL have shown growth-inhibitory effects [39]. ɑ-Pinene, the main component of *Chrysanthemum boreale*, has shown an inhibitory effect at 0.25 mg/mL or higher [40], and rosemary (Rosemary-R), lemon grass (LG), floral petal (Floral-FR), and orange (Orange-O) essential oils have shown antibacterial effects at 0.156 mg/mL or higher [41]. The inhibition of biofilm formation by *S. mutans* was confirmed here by adhesion inhibition on dishes, resin-based teeth, and S-HA. As a result of SEM and confocal observation, a similar inhibitory activity with the 0.1% NaF positive control could be observed with AREO at 0.04 mg/mL. The antibacterial activity was stronger than that of ɑ-pinene from *C. boreale* [40] at a concentration of 0.5 mg/mL or higher, of propolis at a concentration of 0.2 mg/mL or higher [17], and of cypress oil at a concentration of 0.1 mg/mL or higher [38]. Similar to 0.1% NaF (the main component of toothpaste), at a concentration of 2 g/mL or higher of continentalic acid, a single component of *Aralia elata*, and at a concentration of 4 µg/mL or higher of kaurenoic acid from *A. elata*, the activity was lower than the biofilm formation inhibitory effect, which seemed to be the result of a single active ingredient [42,43]. The biofilm formation inhibitory effect was observed as an antibacterial effect of AREO on *S. mutans*. In terms of the genetic effects, considering that the expression of genes related to bacterial adhesion (*gbpB* and *spaP*) was significantly reduced compared to that of genes related to extracellular polysaccharide synthesis (*gtfB*, *gtfC*, and *gtfD*), AREO appeared to have an inhibitory effect on biofilm formation by inhibiting the formation of *S. mutans* attachment to the tooth surface. Propolis, which has a biofilm inhibitory effect, has also been reported to affect the expression of bacterial adhesion-related genes (*gbpB* and *spaP*) [17], and *Chamaecyparis obtusa* oil has been reported to affect the synthesis of extracellular polysaccharides and the expression of genes related to bacterial adhesion [38]. AREO seems to suppress bacterial growth and biofilm formation by inhibiting the expression of genes related to tooth surface adhesion.

The GC and GC-MS analyses in this study confirmed that estragole (88.69%), *β*-caryophyllene (2.56%), and limonene (2.29%) were present in AREO. The component analysis of AREO was analyzed and showed a similar trend to those reported in previous studies, and the antibacterial activity of estragole, the main component, has been reported in several studies. The antibacterial activity of *S. mutans*, the causative agent of dental caries, was confirmed, and the effect of inhibiting dental caries seemed to be due to the inhibition of biofilm formation, which was presumed to be due to the effect of suppressing the expression of genes related to adhesion to tooth surfaces. In the future, the efficacy of the components of AREO may require further research through a pure refining process.

## 4. Materials and Methods

### 4.1. Plant Materials and Essential Oils

Fresh leaves of *A. rugosa* were collected from a local farm in Yusong-Gu, Daejeon Metropolitan City, Republic of Korea, in late August 2021. The samples were authenticated by Professor Byung-Kil Choo (Department of Crop Agriculture and Life Science, Jeonbuk National University, Jeonbuk, Republic of Korea). Voucher specimen (AR-202105) was stored in the Laboratory of Fermentation Technology (Professor Myung-Kon Kim, Jeonbuk National University, Jeonbuk, Republic of Korea). The collected sample was kept in an airtight plastic container and stored in a cold room (4 °C) for 2 days until use. The leaves (1 kg) of *A. rugosa* were mechanically ground and extracted by distillation for 3 h using a Clevenger-type apparatus. The AREO used was a pale yellow oil with a yield of 1.02% based on the fresh weight of the plant. The AREO was stored in a cryogenic freezer (−70 °C) to minimize the loss of volatile compounds.

### 4.2. Strains and Culture

The strain used in this experiment was inoculated with *S. mutans* ATCC 25175 at a concentration of 1 × 10⁸ CFU/mL in brain heart infusion (BHI, BD, Sparks, MD, USA) broth, and after adding samples by concentration, it was then incubated at 37 °C for 24 h. Then, the optical density was measured at 550 nm using a spectrophotometer (Spectra Max 250, Molecular Devices Co., Menlo Park, CA, USA). Moreover, 0.1% NaF was used as a positive control. The experiment was repeated three times.

### 4.3. Adhesion to Saliva-Coated Hydroxyapatite Beads

The saliva secreted by stimulation with paraffin wax from a healthy adult male was collected in a cooled beaker. The collected saliva was centrifuged (12,000 rpm, 4 °C, 15 min), the supernatant was collected and treated at 60 °C for 30 min to inactivate the enzymes, and then used while storing at −20 °C. Hydroxyapatite beads (Bio-Rad Lab., Hercules, CA, USA) were washed five times with distilled water to remove small particles and dried at 37 °C before use. The saliva was coated on the beads by treatment with 30 mg of the dried hydroxyapatite beads with 1 mL of saliva at 37 °C for 60 min. Then, after washing the beads three times with 0.1 M potassium phosphate buffer (KPB, pH 7.0), AREO was added at each concentration. *S. mutans* was added at a concentration of 1 × 10^7^ CFU/mL and adhered to saliva-coated hydroxyapatite (S-HA) beads for 90 min in a shaking incubator at 37 °C. Then, after washing three times with 0.1 M KPB (pH 7.0), bacteria adhered to the S-HA were removed using an ultrasonic device (50 W, 30 s). Then, the bacterial solution was diluted and spread on a Mitis Salivarius Agar plate (BD, Sparks, MD, USA) and cultured for 24 h at 37 °C. Next, the number of colonies was counted [44]. AREO was not added to the control group.

### 4.4. S. mutans Biofilm Formation

After adding BHI liquid medium and AREO to a 35 mm dish, the bacteria were inoculated at a concentration of 5 × 10^5^ CFU/mL. After 24 h of culture in a 37 °C incubator, all of the supernatant was removed. Each dish was washed with 1.5 mL of distilled water. After staining with 0.1% safranin for 30 s, they were washed twice with distilled water, dried, and photographed [44].

### 4.5. S. mutans Biofilm Formed on the Surface of the Artificial Teeth

After adding BHI liquid medium and AREO to artificial teeth (Endura, Shofu Inc., Kyoto, Japan), the bacteria were inoculated at a concentration of 5 × 10^5^ CFU/mL. After culturing for 24 h in a 37 °C incubator, all the filtrate was removed. Each artificial tooth was washed with 1.5 mL of distilled water. After staining with 0.1% safranin for 30 s, it was washed twice with distilled water, dried, and photographed [45].

### 4.6. Measurement of S. mutans Biofilm Using a Scanning Electronic Microscope

After adding BHI liquid medium and AREO to a 35 mm dish, the bacteria were inoculated at a concentration of 5 × 10^5^ CFU/mL. After 24 h of culture in a 37 °C incubator, all the supernatant was removed. Each dish was washed with 1.5 mL of distilled water. The bacteria were then fixed for 24 h in a 2.5% glutaraldehyde solution (in a 0.1 M sodium cacodylate buffer, pH 7.2, 4 °C). Starting with 70% ethanol, washing and dehydration were performed by increasing the concentration to 80%, 95%, and 100%. After freeze-drying, the bacteria were coated with gold and photographed by scanning electron microscopy (SEM) [45].

### 4.7. Acid Production of S. mutans

After adding the sample to the BHI liquid medium containing 1% glucose, the bacteria were inoculated to a concentration of 1 × 10^8^ CFU/mL. After culturing for 24 h in an incubator at 37 °C, absorbance was measured at 550 nm using an ELISA reader (Molecular Devices Co., San Jose, CA, USA) based on the BHI broth. The pH was measured using a pH meter (Mettler-Toledo, Schwerzenbach, Switzerland) to observe the inhibitory effect on acid production. The control underwent the same process without adding the sample [46].

### 4.8. Real-Time PCR Analysis

Real-time PCR was performed to evaluate the effect of *A. rugosa* on *S. mutans* gene expression. cDNA was synthesized by isolating total RNA from *S. mutans* cultured in samples treated with different concentrations of the extract. Amplification was performed using a StepOnePlus Real-time PCR system with QPCR SYBR Green Mixes (Applied Bio system, Foster City, CA, USA). In addition, 16S rRNA was used as a control. Primer pairs have been described in a previous study [42]. Primer pairs are listed in Table 3. Assays were performed in duplicate on at least two independent RNA samples.

### 4.9. GC and GC-MS Analyses

GC analysis was performed on a Hewlett–Packard model 6890 series gas chromatograph with a flame ionization detector and a split ratio of 1:30 using a DB-5HT-fused silica capillary column (30 m × 0.32 mm, i.d., 0.25 μm film thickness). The column temperature was programmed from 40 °C to 230 °C at 2 °C/min and then kept constant at 230 °C for 20 min. The injector and detector temperatures were 230 °C and 250 °C, respectively. The carrier gas was nitrogen, with a flow rate of 0.80 mL/min. Peak areas were measured by electronic integration, and the relative amounts of the individual components were based on the peak areas.

GC-MS analysis was performed on an Agilent Technologies 7890A GC and 5975C mass selective detector operating in the EI mode at 70 eV and fitted with a DB-5MS-fused silica capillary column (30 m × 0.25, i.d., 0.25 μm film thickness). The column temperature was programmed from 40 °C to 230 °C at 2 °C/min and then kept constant at 230 °C for 20 min. The injector and ion source temperatures were both 250 °C. The carrier gas was helium at a flow rate of 1.0 mL/min. The identification of individual components was based on comparisons of mass spectra with the NIST/NBS mass spectral database and retention indices with data from previous studies [21]. Linear retention indices were calculated against those of an n-paraffin (C_6_∼C_26_) series [47].

### 4.10. Statistical Processing

The experiment was repeated three times, and the obtained results were presented as the average and standard deviation (SD) using the statistical program SPSS (ver. 10.0). At the *p* = 0.05 level, the average value of the experimental group and the control group was verified by an independent sample *t*-test.

## 5. Conclusions

This study demonstrated the antibacterial and antibiofilm effects of AREO on *S. mutans.* In addition, the effects on the expression of genes related to biofilm formation of *S. mutans* were investigated. Therefore, AREO is expected to be a promising agent to prevent dental caries. AREO is mainly composed of estragole, β-caryophyllene, and limonene, which may be responsible to be an antibacterial component, and inhibit growth and acid secretion in planktonic cultures. In this study, we focused on the overall anti-cariogenic potential of AREO and identified its major components. Although the individual inhibitory effects of the compounds such as limonene, estragole, and beta-caryophyllene were not tested in this study, future research will explore their specific roles in growth inhibition, adhesion, biofilm formation, and acid production. This initial study is valuable in providing a holistic understanding of AREO’s anti-cariogenic properties and its potential as a natural agent against dental biofilm. To our knowledge, this is the first report that AREO shows anti-cariogenic properties. However, despite the novelty and significance of this study, there are some limitations. One limitation is that the biofilm formation and the gene expression levels do not correspond, and another is that the antimicrobial effects of the major compounds need to be further investigated in future studies.

## Figures and Tables

**Table 1:** The pH changes in *S. mutans* cultures incubated with different concentrations of AREO.

Concentration (mg/mL)	pH (Before Incubation)	pH (After Incubation)
Control	7.38 ± 0.05	5.41 ± 0.05
0.01	7.38 ± 0.05	5.48 ± 0.10
0.02	7.38 ± 0.05	5.74 ± 0.00 *
0.03	7.38 ± 0.05	6.54 ± 0.11 *
0.04	7.38 ± 0.00	7.19 ± 0.05 *
NaF (0.1%)	7.38 ± 0.00	7.37 ± 0.00 *

Data (pH) are represented as the mean ± SD. * *p* < 0.05 as compared with the control group after incubation.

**Table 2:** AREO constituents.

Compound	Retention Index		Peak Area (%)
	Calculated ^1)^	Literature ^2)^	
2-Ethyl furan	712	713	t
*n*-Hexanal	799	800	t
*trans*-2-Hexenal	862	862	0.06
*cis*-3-Hexen-1-ol	867	858	t
α-Pinene	930	939	t
Camphene	950	953	0.08
Benzaldehyde	958	961	0.12
Sabinene	967	971 ^3)^	t
1-Octen-3-ol	985	978	0.45
3-Octanone	987	986	0.08
Myrcene	991	991	0.05
Limonene	1031	1031	2.29
Benzyl alcohol	1037	1032	0.06
Phenylacetaldehyde	1039	1043	0.07
Linalool	1102	1098	t
1-Octen-3-yl acetate	1121	1119	0.24
*trans*-Isopulegone	1181	1175	0.13
Estragole	1219	1196	88.69
Chavicol	1287	1265 ^3)^	1.05
Isopiperitenone	1292	1282 ^3)^	0.08
*trans*-Anethole	1300	1285	t
*p*-Vinylguaiacol	1323	1324	t
Eugenol	1366	1356	0.22
Unidentified	1379	-	0.24
β-Bourbonene	1383	1384	0.05
*cis*-Jasmone	1395	1394	0.05
Methyl eugenol	1404	1401	0.06
β-Caryophyllene	1424	1418	2.56
α-Humulene	1447	1454	0.12
Germacrene D	1479	1480	0.80
Bicyclogermacrene	1493	1494	0.35
*trans*-Methyl isoeugenol	1505	1495 ^3)^	0.07
*trans,trans*-α-Farnesene	1508	1508	t
δ-Cadinene	1521	1524	0.08
Spathulenol	1570	1576	0.07
Caryophyllene oxide	1573	1581	0.06
τ-Cadinol	1627	1625	t
τ-Muurolol	1635	1641	0.05
α-Cadinol	1648	1653	0.07
6,10,14-Trimethylpentadecan-2-one	1842	1843	t
Benzyl salicylate	1883	1876 ^3)^	t
Oleic acid	2113	2115 ^3)^	0.27
Total			98.58

^1)^ Retention indices on nonpolar DB-5HT fused silica capillary column. ^2)^ Adams, Identification of essential oil components by gas chromatography/mass spectroscopy [21]. ^3)^ NIST (National Institute of Standards and Technology) Chemistry webbook, SRD (Standard Reference Database) no. 69, 2023. t: trace (peak area less than 0.05%).

**Table 3:** Oligonucleotide primers used in this study.

Genes *	Genes Description	Primer Sequences (5′-3′)	
*16S rRNA*	Normalizing internal standards	Forward	CCTACGGGAGGCAGCAGTAG
		Reverse	CAACAGAGCTTTACGATCCGAAA
*gtfB*	Glucosyltransferase B (gtfB)	Forward	AGCAATGCAGCCAATCTACAAAT
		Reverse	ACGAACTTTGCCGTTATTGTCA
*gtfC*	Glucosyltransferase SI (gtfC)	Forward	GGTTTAACGTCAAAATTAGCTGTATTAGC
		Reverse	CTCAACCAACCGCCACTGTT
*gtfD*	Glucosyltransferase-I (gtfD)	Forward	ACAGCAGACAGCAGCCAAGA
		Reverse	ACTGGGTTTGCTGCGTTTG
*brpA*	Biofilm-regulation protein	Forward	GGAGGAGCTGCATCAGGATTC
		Reverse	AACTCCAGCACATCCAGCAAG
*gbpB*	Glucan-binding protein	Forward	ATGGCGGTTATGGACACGTT
		Reverse	TTTGGCCACCTTGAACACCT
*relA*	Guanosine tetra (penta)-phosphate synthetase	Forward	ACAAAAAGGGTATCGTCCGTACAT
		Reverse	AATCACGCTTGGTATTGCTAATTG
*spaP*	Cell surface antigen	Forward	GACTTTGGTAATGGTTATGCATCAA
		Reverse	TTTGTATCAGCCGGATCAAGTG
*vicR*	Two-component system regulatory	Forward	TGACACGATTACAGCCTTTGATG
		Reverse	CGTCTAGTTCTGGTAACATTAAGTCCAATA

* Based on the NCBI *S. mutans* genome database.

## Data Availability

The data that support the findings of this study are available from the corresponding author upon reasonable request.
